# Delusional infestation in healthcare professionals: Outcomes from a multi‐centre case series

**DOI:** 10.1002/ski2.122

**Published:** 2022-11-28

**Authors:** John Frewen, Peter Lepping, Jonathan M. R. Goulding, Stephen Walker, Anthony Bewley

**Affiliations:** ^1^ Department of Dermatology Royal Devon and Exeter NHS Foundation Trust Exeter Devon UK; ^2^ Department of Psychiatry Betsi Cadwaladr University Health Board Wrexham UK; ^3^ Centre for Mental Health and Society Bangor University Wrexham UK; ^4^ Department of Dermatology University Hospitals Birmingham NHS Foundation Trust Birmingham UK; ^5^ London School of Hygiene and Tropical Medicine London UK; ^6^ Hospital for Tropical Diseases and Department of Dermatology University College London Hospitals NHS Foundation Trust London UK; ^7^ Department of Dermatology Barts Health NHS Trust London UK

## Abstract

Delusional infestation (DI) describes an unwavering fixed belief of infestation with pathogens, despite a lack of medical evidence supporting this. Effective management of DI with antipsychotics is made challenging by the fixed belief that the condition is an infestation or infection rather than a mental illness. A case series of individuals diagnosed with DI included 11% who were healthcare professionals (HCPs). We sought to characterise a cohort of HCPs who presented with DI in the UK. The case notes of HCPs diagnosed with DI at specialist clinics between 2015 and 2019 were reviewed. Demographic and clinical data were obtained. Twelve HCPs were identified out of a total of 381 individuals diagnosed with DI. Median age was 52.5 (IQR = 14.5) years. 75% (*n* = 9) were women. Ten individuals had primary DI, whilst two had secondary DI (one to recreational drug use, one to depression). Four individuals (33%) engaged with antipsychotic treatment. Two responded well, both had secondary DI. Of the two individuals with primary DI who engaged, one did not respond to antipsychotic medication and the other was unable to tolerate two antipsychotic drugs. In Primary DI (*n* = 10), the rate of adherence was lower at 20% (*n* = 2). In DI, high engagement and adherence rates to treatment have been reported in specialist centres. Improvement has been reported as high as 70%–75%. This indicates that a large proportion of individuals who adhere to treatment appear to derive benefit. In this series, engagement with treatment by HCPs with primary DI was low at 20%, and improvement was only achieved in individuals with secondary DI. Mental illness‐related stigma, feelings of distress and difficulty forming therapeutic relationships with a professional peer are significant challenges. Developing rapport is key to treatment success in DI. In HCPs this may be suboptimal due to these negative feelings, resulting in lower engagement. A diagnosis of DI in a HCP may raise concerns regarding fitness to practise. An assessment of the impact of DI and the potential to interfere with professional duties warrants consideration. We highlight the occurrence of DI in HCPs, and the apparent lower engagement with treatment in this cohort.

1

**What's already known about this topic?**

- Delusional infestation (DI) is challenging to manage and early use of anti‐psychotic medication is associated with improved outcomes.
- Healthcare professionals with DI have not previously been characterized.

**What does this report/study add?**

- Healthcare professionals with DI had a low rate of engagement with antipsychotic treatment.
- Engagement was achieved in healthcare professionals with secondary DI but not those with primary DI.
- We highlight factors which may explain low engagement with and low response to treatment in this cohort.

## 2 INTRODUCTION

Delusional infestation (DI) describes a fixed belief of infestation with pathogens, held with delusional intensity despite a lack of medical evidence supporting this.^1^ Individuals present most frequently to dermatology (53%), but also to other specialties such as internal medicine, emergency medicine infectious diseases and psychiatry.^2^ Effective management of DI with antipsychotics is made challenging by the fixed belief that the condition is an infestation or infection rather than a psychiatric illness.

A US population‐based prevalence survey reported an incidence of DI of 1.9 (95% CI, 1.5–2.4) per 100 000 person‐years.^3^ An estimated prevalence in Germany of 83.2 per 1 million of the population was calculated from surveys of clinics.^4^ The point prevalence of DI is 1.48 per million dermatology outpatient attendees in the UK.^5^ DI is more frequently diagnosed in women.^2^, ^6^

The prevalence of psychiatric disorders may be higher amongst healthcare professionals (HCP) than the general population.^7^ A retrospective case series of individuals diagnosed with DI at a tertiary care academic medical centre in the United States included 16 individuals of 145 (11%) who were HCPs.^2^ The authors did not comment on the burden of disease, or the likelihood of engagement with treatment in this cohort. We sought to characterise a cohort of HCPs who presented with DI to specialist clinics in the UK and describe their rates of engagement with and efficacy of treatment.

## 3 REPORT

The case notes of HCPs diagnosed with DI at specialist outpatient clinics in Birmingham, Liverpool and London during the 5‐year period of 2015–2019 were reviewed. HCPs were defined as individuals who performed clinical duties requiring direct interaction with health service users. The following data were obtained: age, sex, type of DI (primary or secondary), treatment engagement and response, complaints made, number of consultations per individual, and presentation to other centres. Treatment engagement was defined as adherence to prescribed antipsychotic medication until at least the next follow‐up appointment.

Three hundred and eighty one individuals were diagnosed with DI, of which 12 were HCPs (3%). Median age was 52.5 (IQR = 14.5) years. Seventy‐five percent (*n* = 9) were women. The HCP disciplines encountered were medicine, nursing, psychotherapy, dentistry, and paramedic sciences. Ten individuals had primary DI, whilst two had secondary DI (one associated with recreational drug use, one with depression). Four individuals (33%) engaged with antipsychotic treatment. Two responded well, both of whom had secondary DI. These two individuals were treated by addressing both the underlying cause (abstaining from recreational drug use, and sertraline for depression respectively) in addition to risperidone. Of the two individuals with primary DI who engaged, one did not respond to antipsychotic medication and the other was unable to tolerate two antipsychotic drugs. The proportion of those with primary DI (*n* = 10) who were adherent was lower at 20% (*n* = 2).

The number of appointments per case ranged from 1 to 7. Four patients (33%) visited other institutions for consultation regarding the same condition. Three patients (25%) made formal complaints.

## 4 DISCUSSION

Adherence to treatment by individuals with psychiatric illnesses is variable and challenging.^8^ For major mental illnesses, the non‐adherence rates vary between 44% and 56%.^8^

In DI, high engagement and adherence rates (48%–95%) to treatment have been reported in specialist centres.^9^, ^10^, ^11^ Longer duration of untreated disease is associated with poorer outcome.^12^ Improvement (partial or complete remission) has been reported as high as 70%–75%.^1^, ^10^ This indicates that a large proportion of individuals who adhere to treatment appear to derive benefits. In this series, engagement with treatment in HCPs with primary DI was low at 20%, and improvement was only achieved in individuals with secondary DI.

The factors explaining low engagement and response to treatment in HCPs with DI have not previously been explored. Medication non‐adherence in psychiatric illness is associated with factors including age over 60 years, busy workload, a negative attitude towards medication, lack of insight, and a perception of being stigmatised by families and health professionals.^8^ Mental illness‐related stigma is well recognized in healthcare.^8^, ^13^ Stigmatization by doctors in the UK is associated with a lower likelihood of doctors seeking support from colleagues or their GPs.^14^ It is possible that when HCPs with DI are recommended antipsychotic treatment, their pre‐existing knowledge of drug classes and indications may lead to resistance to engagement, through medication recognition. However, the increased accessibility of information about medications may make the specialist knowledge of HCPs less relevant to the seemingly lower rates of engagement with treatment.

It is recognized that when HCPs adopt the role of patients, feelings of distress, vulnerability, loss of control, humiliation, and judgement occur, as well as a perceived difficulty with forming therapeutic relationships with professional peers.^15^, ^16^, ^17^ Developing rapport early is key to treatment success in DI. In HCPs this may be suboptimal due to these negative feelings, resulting in lower engagement. Recognising and addressing these distressing emotions is key to optimising rapport.

Stress‐related mental disorders such as depression and post‐traumatic stress disorder are more prevalent in HCPs, as exposure to a large number of environmental and psychosocial stressors is common.^18^, ^19^ Suicide rates are higher amongst nurses, doctors and paramedics compared to the general population.^20^, ^21^, ^22^ HCPs often fear that their mental health diagnosis will not remain confidential,^14^ which may reduce help‐seeking behaviour, and perpetuate their illness. Most individuals with DI do not fulfil criteria for treatment under the Mental Health Act, but a diagnosis of DI in a HCP may raise concerns regarding fitness to practise.^23^ An assessment of the impact of DI and the potential to interfere with professional duties needs to be considered. The General Medical Council states that a question of fitness to practise could arise if a condition may affect a doctors' conduct or clinical care they provide, and they are not following treatment, advice, or engaging with local support.^24^ The Nursing and Midwifery Council assesses fitness to practice for individuals with long term, untreated, or unacknowledged mental or physical health conditions, in which there is a risk to public protection.^25^ The association of reporting mental illness and potential disciplinary measures can be a further hindrance to seeking help. Career implications are cited by doctors as the main reason for not disclosing their mental illness.^14^ This fear serves to further stigmatize mental illness amongst HCPs, and potentially reduce engagement with treatment. We consider that the lower engagement rate of our cohort with treatment compared to previously published data on DI, may relate to these factors of stigmatization, negative emotions adopting the patient role, additional mental health disorders, and fear of disclosure to professional bodies.

HCPs with DI are challenging to manage. We highlight the lower engagement and response to treatment in this cohort. Engagement continues to be critically important to successful outcomes. Strategies to engage HCPs with DI may need to be different from standard strategies, namely greater consultation time and better understanding of ways of utilising the measures which we have reported can work.^9^, ^10^, ^11^ If HCPs with DI do not engage, continued efforts to develop sufficient rapport may be challenging with the potential risk that their mental illness may affect their fitness to practice. The work of engaging individuals with DI who are HCPs is likely to need the development of newer techniques, emphasising the need for further research in this area. At present, evidence supports the use of antipsychotic medication for treating DI, however perhaps future research may examine the role of psychotherapy in this group. The development of novel strategies may improve our management of other individuals with DI.

## AUTHOR CONTRIBUTIONS

**John Frewen:** Formal analysis (lead); Investigation (lead); Writing – original draft (equal); Writing – review & editing (equal). **Peter Lepping:** Formal analysis (supporting); Investigation (supporting); Supervision (supporting); Writing – review & editing (supporting). **Jonathan M. R. Goulding:** Formal analysis (supporting); Investigation (supporting); Supervision (supporting); Writing – review & editing (supporting). **Stephen L. Walker:** Formal analysis (supporting); Investigation (supporting); Supervision (lead); Writing – original draft (equal); Writing – review & editing (equal). **Anthony Bewley:** Formal analysis (supporting); Investigation (supporting); Supervision (supporting); Writing – review & editing (supporting).

## CONFLICT OF INTEREST

The authors declare no conflict of interest.

## Data Availability

Data available on request due to privacy/ethical restrictions.
